# Organic amendments boost maize yield (*Zea mays* L.) in karst soils via a hierarchical process driven by soil phosphorus enhancement and microbial-mediated nutrient cycling

**DOI:** 10.3389/fpls.2026.1782544

**Published:** 2026-04-10

**Authors:** Rong Yang, Jingwei Zhu, Yungui Zhang, Yanxia Liu, Zhihong Li, Heng Zhang, Qiongxiang Li, Xinxiu Wang, Xi Chen, Di Chen, Qingli Liu

**Affiliations:** 1 State Key Laboratory of Efficient Utilization of Arable Land in China, Institute of Agricultural Resources and Regional Planning, Chinese Academy of Agricultural Sciences, Beijing, China; 2 Guizhou Tobacco Science Research Institute, Guiyang, China; 3 Institute of Environment and Sustainable Development in Agriculture, Chinese Academy of Agricultural Sciences, Beijing, China

**Keywords:** functional genes, karst yellow soil, maize yield, microbial community, organic amendments, soil nutrients

## Abstract

**Introduction:**

Sustainable food production in fragile karst landscapes requires moving beyond input-intensive agriculture.

**Methods:**

This study investigated how long-term organic amendments affected maize yield, using a 15-year field trial on karst yellow soil. Integrating soil analysis, metagenomics, and causal modeling, revealed that adding farmyard manure or bio-organic fertilizer to mineral NPK increased yield by 12.08% and 11.48%, respectively, and improved key soil properties, most notably available phosphorus.

**Results:**

Organic inputs shifted the soil microbiome toward copiotrophic taxa and enriched genes for organic matter decomposition and phosphorus mobilization. However, statistical modeling revealed that these biological changes did not directly drive yield. Instead, the primary pathway was hierarchical: amendments first enhanced the soil’s chemical habitat, which then directly boosted crop growth while simultaneously shaping the microbial community and its functional potential. The interaction of soil, microbes, and genes together explained 81% of the yield variation.

**Discussion:**

Our findings demonstrate that in phosphorus-limited karst soils, organic amendments act foremost as soil conditioners. Microbial processes, though crucial, are secondary mediators that translate improved soil conditions into efficient nutrient cycling. Therefore, sustainable intensification in these vulnerable agroecosystems should prioritize managing soil health over directly targeting microbial processes.

## 1 Introduction

The contemporary epoch of agricultural intensification confronts a profound biogeochemical paradox: while mineral fertilizer application has been instrumental in achieving global food security to a large extent for decades, its persistent overuse now constitutes a primary driver of terrestrial ecosystem degradation (Rockström et al., 2017). This trajectory has precipitated a cascade of pedospheric disturbances including, but not limited to, systemic soil acidification through proton release during nitrification and ammonium uptake, phosphorus fixation complexes in alkaline and calcareous soils, depletion of soil organic matter (SOM) pools due to reduced carbon inputs, and disruption of microbial-mediated nutrient cycling networks (Dai et al., 2018; Xu et al., 2024). The resultant decline in soil multifunctionality—encompassing nutrient buffering capacity, water retention efficiency, and biodiversity maintenance—creates a pernicious feedback loop wherein degraded soils necessitate increased fertilizer inputs to maintain yields, thereby accelerating environmental degradation (Lal, 2020). This conundrum necessitates a paradigmatic shift from input-intensive agriculture towards ecological intensification strategies that reconcile productivity with planetary boundaries. Within this context, organic amendments emerge as critical levers for rebuilding soil capital, not merely as nutrient sources but as architects of soil habitat complexity (Luo et al., 2018; Ma et al., 2024).

Organic amendments, spanning the spectrum from traditional farmyard manure to engineered bio-organic formulations, function as integrative soil conditioners through three synergistic mechanisms: chemical stabilization of soil structure, enrichment of nutrient pools of soil, and ecological stimulation of soil biota (Qaswar et al., 2020; Mustafa et al., 2022; Imran et al., 2023). The incorporation of complex organic matrices enhances soil porosity through aggregate formation mediated by fungal hyphae and microbial exopolysaccharides, while simultaneously increasing cation exchange capacity through the formation of organo-mineral complexes (Tisdall and Oades, 1982). Crucially, these amendments serve as slow-release nutrient banks, mitigating the leaching losses characteristic of soluble fertilizers through controlled mineralization kinetics mediated by extracellular enzyme activities (Geisseler and Scow, 2014). Beyond these material contributions, organic inputs fundamentally restructure soil microbial metabolic networks by shifting the competitive landscape from oligotrophic strategists adapted to nutrient scarcity toward copiotrophic assemblages optimized for resource acquisition in enriched environments (Fierer et al., 2007; Chen et al., 2025). However, the efficacy of this ecological engineering is profoundly mediated by geogenic constraints, particularly in karst landscapes where pedogenesis occurs on carbonate bedrock. Karst soils, exemplified by the Haplic Alisols of Southwest China, present a unique convergence of challenges: shallow solum depth, high calcium carbonate content inducing phosphorus fixation, rapid hydraulic conductivity promoting nutrient leaching, and low native organic matter reserves (Li et al., 2021; Gong et al., 2022). In these geochemically constrained systems, the conventional fertilizer paradigm proves particularly detrimental, while organic amendment strategies may elicit unexpected outcomes due to complex interactions between imported organic matter and the native carbonate matrix (Yan et al., 2021).

The transformation of organic amendments into plant-available nutrients constitutes a sophisticated bioreactor mediated by soil microbiomes, yet the precise mechanisms governing this transformation remain partially unresolved within a “black box” of biological complexity (Philippot et al., 2024). Contemporary research has transcended taxonomic inventories to interrogate the functional genomics of nutrient cycling, revealing that microbial communities respond to organic inputs through transcriptional and translational reprogramming of metabolic pathways (Zhu et al., 2024). Metagenomic analyses have elucidated how carbon complexity in organic amendments leads to the selective enrichment of specific carbohydrate-active enzyme (CAZyme) profiles, how nitrogenous compounds shape the relative abundance of nitrification versus denitrification genes, and how phosphorus speciation influences the expression of phosphatase genes and phosphate transporter systems (Zeng et al., 2022; Chen et al., 2024). Particularly in karst ecosystems, where phosphorus exists predominantly in sparingly soluble calcium-phosphate complexes, the microbial genetic repertoire for organic phosphorus mineralization and inorganic phosphate solubilization is of critical importance (Helfenstein et al., 2024). However, a persistent epistemological gap remains between documenting these molecular responses and establishing causal linkages to agronomic outcomes. The fundamental question persists: are observed microbial and genomic shifts direct drivers of crop productivity, or merely epiphenomenal correlates of improved soil chemical conditions? This ambiguity is compounded by the recognition that microbial functional potential (as revealed by gene abundance) is often decoupled from actual process rates due to post-transcriptional regulation and substrate limitation (Jing et al., 2020). Resolving this requires experimental designs that simultaneously quantify soil chemical parameters, microbial community structure, functional gene abundance, and plant performance within a unified analytical framework.

Current understanding of organic amendment effects remains fragmentary across three critical dimensions: temporal (short-term responses dominate the literature), systemic (compartmentalized studies of chemistry, biology, or yield), and mechanistic (correlative rather than causal inference) (Liu et al., 2025). Few investigations have pursued the longitudinal trajectory of soil-microbe-plant systems under sustained organic management, particularly in inherently vulnerable edaphic environments like karst landscapes. This knowledge fragmentation impedes the development of predictive models for sustainable intensification in precisely those regions where food security is most precarious (D'Ettorre et al., 2024). To address these interconnected gaps, we implement an integrative research framework combining long-term field experimentation, deep molecular profiling, and multivariate causal modeling. Our investigation leverages a 15-year continuous fertilization trial (initiated in 2008) in the karst plateau of Guizhou Province, China, incorporating four management regimes: an unamended control (CK), mineral NPK fertilization (NPK), NPK plus farmyard manure (FNPK), and NPK plus bio-organic fertilizer (MNPK). Through this design, we carried out three synergistic objectives: (1) To quantify the legacy effects of long-term organic amendments on the complete soil chemical matrix, with particular emphasis on phosphorus speciation and bioavailability in calcareous systems; (2) To characterize concomitant transformations in microbial community architecture and functional gene networks governing carbon, nitrogen, and phosphorus metabolism using shotgun metagenomic sequencing; and (3) To deconvolute the hierarchical pathways through which management signals propagate through the soil-plant system using variance partitioning analysis (VPA) and partial least squares path modeling (PLS-PM). By synthesizing pedological, genomic, and agronomic data streams, this study aims to transition from phenomenological description to mechanistic understanding, ultimately generating a predictive framework for sustainable nutrient management in Earth’s most vulnerable agroecosystems.

## 2 Materials and methods

### 2.1 Site description and experimental design

The long-term experiment was conducted at the Soil Quality Monitoring Station in Kaiyang County, Guizhou Province, China (107°06′40.8″E, 26°52′24.8″N). The site has a northern subtropical humid monsoon climate. A fertilization experiment was initiated there in 2008, following a typical upland crop rotation dominated by maize and tobacco. A randomized complete block design was employed with four treatments: (1) an unfertilized control (CK); (2) mineral fertilizer only (NPK); (3) mineral fertilizer plus farmyard manure (FNPK); and (4) mineral fertilizer plus bio-organic fertilizer (MNPK). Each plot measured 144 m^2^ and was isolated by concrete barriers to prevent lateral nutrient movement.

The mineral fertilizers used were ammonium phosphate, superphosphate, and potassium sulfate. For the NPK, FNPK, and MNPK treatments, mineral fertilizers were applied at rates of 120 kg N ha^-1^, 60 kg P_2_O_5_ ha^-1^, and 60 kg K_2_O ha^-1^ during the maize growing season (April to September), with phosphorus inputs calculated from both ammonium phosphate and superphosphate. The farmyard manure consisted of composted cattle manure (N 1.4%; P_2_O_5_ 0.4%; K_2_O 2.1%) and was applied at 7,500 kg ha^-1^ for the FNPK treatment. The bio-organic fertilizer, with a composition of N 2.6%, P_2_O_5_ 2.2%, and K_2_O 2.8% and containing 1×10^8^ *Bacillus* spores g^-1^, was applied at 750 kg ha^-1^ for the MNPK treatment. The maize variety was the locally dominant cultivar Yuhaodan 5, with a planting density of 45,000 plants ha^-1^. Maize was sown in April 2023 and harvested in September 2023. All field management practices followed standard local agronomic protocols. Fertilizers were applied as one basal application and two topdressings, and weeds were controlled manually without the use of herbicides.

### 2.2 Sample collection and processing

Soil samples were collected in September, 2023 immediately after harvest. From each plot, three composite samples were obtained from the 0–20 cm tillage layer using a multi-point random sampling approach. After removing roots, stones, and visible debris, the soil was sieved through a 2.0 mm mesh. Each composite sample was then divided into two aliquots: one was flash-frozen in liquid N_2_ and stored at −80 °C for subsequent metagenomic analysis, and the other was air-dried for chemical characterization. Concurrently, ten representative maize plants were randomly selected per net plot for grain yield determination.

### 2.3 Laboratory analysis

#### 2.3.1 Soil chemical properties

Soil pH was measured potentiometrically in soil water suspension (1:2.5 w/v). Soil organic matter (SOM) was determined using the potassium dichromate oxidation–titration method. Cation exchange capacity (CEC) was measured using the cobalt hexamine trichloride extraction method and quantified spectrophotometrically. Alkali-hydrolyzable nitrogen (AN) was determined using the alkali-hydrolysis diffusion–titration method. Available phosphorus (AP) was determined as NaHCO_3_-extractable P using a colorimetric method. Briefly, soil samples were extracted with 0.5 M NaHCO_3_, and the P concentration in the extract was measured using a UV–visible spectrophotometer (Olsen et al., 1954). Available potassium (AK) was determined as NH_4_OAc-extractable K. Soil samples were extracted with 1 M NH_4_OAc, and K concentration was measured using a flame photometer. The above routine soil chemical properties were determined according to the standard procedures described by Bao (2008). Available boron (AvB) was determined using the azomethine-H colorimetric method. Exchangeable calcium (ExCa), exchangeable magnesium (ExMg), available sulfur (AvS), and available micronutrients (Fe, Mn, Cu, Zn, and Mo) were extracted and quantified using Inductively Coupled Plasma Mass Spectrometry (ICP–MS) according to standard protocols (Melaku et al., 2005; Bao, 2008).

#### 2.3.2 Metagenomic sequencing, gene prediction, and functional annotation

Total genomic DNA was extracted from soil samples, and DNA quality was assessed via agarose gel electrophoresis and quantified using a Qubit fluorometer. DNA libraries were constructed by fragmenting 1 μg of genomic DNA to approximately 350 bp using a Covaris ultrasonic disruptor, followed by end repair, A-tailing, adapter ligation, and PCR amplification. Library quality was verified using an Agilent 2100 Bioanalyzer and quantitative PCR. Sequencing was performed on the Illumina HiSeq platform (PE 150) by Novogene Bioinformatics Technology Co., Ltd. (Beijing, China).

Raw reads were quality-filtered using Readfq to remove adapters and low-quality bases. Host contamination was removed by aligning reads to the host genome using Bowtie2 (Karlsson et al., 2012, 2013). High-quality clean reads were assembled into scaffolds using MEGAHIT (Nielsen et al., 2014). Scaffolds were split into contigs (scaftigs) at ambiguous N bases (Li et al., 2015a). Open reading frames (ORFs) were predicted using MetaGeneMark (Karlsson et al., 2012; Mende et al., 2012) and clustered using CD-HIT to construct a non-redundant gene catalogue (Li and Godzik, 2006; Fu et al., 2012). Clean reads were mapped to this catalogue using Bowtie2 to calculate gene abundance (Li et al., 2014). Taxonomic annotation was performed by aligning unigenes against the NCBI NR database (Bacteria, Fungi, Archaea, and Viruses) using DIAMOND, with taxonomic assignments determined by the Lowest Common Ancestor (LCA) algorithm (Karlsson et al., 2012; Li et al., 2014; Feng et al., 2015).

Functional annotation of carbon, nitrogen, and phosphorus cycling genes was conducted using DIAMOND BLASTP (E-value ≤ 1e^-5^) against the KEGG and CAZy databases. Nitrogen- and phosphorus-related genes were annotated using KEGG, while carbon cycling genes were identified using CAZy. Only the highest-scoring hit for each unigene was retained. Functional gene abundance and gene counts at different hierarchical levels were performed and used for subsequent functional profiling and group comparison analyses.

### 2.4 Statistical analysis

Statistical analyses were conducted in R (v4.2.1). Between-treatment differences in yield, soil properties, and microbial α-diversity (Shannon index) were assessed using the one-way Kruskal-Wallis test, followed by Dunn’s test with Benjamini-Hochberg correction. Microbial β-diversity was examined via NMDS based on Bray–Curtis distances, with community structure differences tested by PERMANOVA. Treatment-specific microbial biomarkers were identified using LEfSe (LDA score > 4.0). Functional gene abundances were normalized against the NPK treatment and visualized using chord and radar diagrams. Stepwise and linear regression identified key yield-associated variables. Variation partitioning analysis (VPA) and redundancy analysis (RDA) quantified the contributions of soil, microbial, and gene factors. Finally, partial least squares path modeling (PLS-PM) was used to elucidate the pathways through which amendments influence yield.

## 3 Results

### 3.1 Effects of organic amendments on maize yield, soil chemical properties, and microbial community structure

Organic amendments significantly (*P* < 0.05) influenced both maize yield and key soil properties (**Table 1**). Compared with the unfertilized control (CK), NPK fertilization increased maize yield by 44.20%. Relative to NPK alone, the addition of organic amendments further boosted yield by 12.08% (FNPK) and 11.48% (MNPK), although no significant difference was observed between the two organic amendment types. Soil chemical characteristics varied markedly among treatments. Notably, the MNPK treatment maintained a soil pH (7.36) statistically similar to the CK (7.35), whereas NPK application caused significant acidification. Both farmyard manure and bio-organic fertilizer significantly (*P* < 0.05) enhanced soil fertility metrics, including cation exchange capacity (CEC), soil organic matter (SOM), alkali-hydrolyzable nitrogen (AN), available phosphorus (AP), and available potassium (AK). For all these parameters, values ranked as follows: MNPK > FNPK > NPK > CK (*P* < 0.05), with the bio-organic fertilizer exerting a stronger positive effect on nutrient retention and SOM accumulation. Regarding micronutrients, compared to the NPK treatment, organic amendments significantly (*P* < 0.05) elevated the levels of exchangeable calcium (ExCa) and available copper (AvCu), zinc (AvZn), and boron (AvB). Divergent effects were observed for other nutrients: FNPK reduced available sulfur (AvS) but increased available iron (AvFe) and manganese (AvMn), while MNPK decreased available molybdenum (AvMo), iron, and manganese. Exchangeable magnesium (ExMg) showed no significant variation across all treatments.

**Table 1:** Effects of different fertilization treatments on maize yield and soil chemical properties.

Parameters	CK	NPK	FNPK	MNPK
Yield (kg ha^−1^)	7387 ± 265.02c	10645 ± 89.08b	11931 ± 249.29a	11868 ± 96.01a
pH	7.35 ± 0.04a	6.76 ± 0.01b	6.75 ± 0.02b	7.36 ± 0.03a
SOM (g·kg^−1^)	43.80 ± 0.89c	47.94 ± 1.01b	48.39 ± 1.11b	50.82 ± 0.92a
CEC (cmol (+) kg^-1^)	22.66 ± 0.39c	22.86 ± 0.51c	24.56 ± 0.23b	26.59 ± 0.30a
AN (mg·kg^−1^)	110.18 ± 1.11d	114.66 ± 0.95c	120.80 ± 1.85b	133.33 ± 0.75a
AP (mg·kg^−1^)	12.17 ± 0.24d	24.31 ± 0.42c	37.53 ± 0.26b	39.91 ± 0.98a
AK (mg·kg^−1^)	66.76 ± 1.38d	109.47 ± 0.70c	160.60 ± 0.21b	197.79 ± 0.69a
ExCa (mg·kg^−1^)	461.29 ± 10.51c	441.87 ± 3.23d	497.34 ± 2.04b	663.35 ± 15.36a
ExMg (mg·kg^−1^)	1960 ± 83.66a	2147 ± 377.81a	2298 ± 189.40a	2113 ± 663.81a
AvS (mg·kg^−1^)	1.12 ± 0.08c	16.63 ± 1.29a	7.37 ± 0.41b	15.85 ± 1.13a
AvFe (mg·kg^−1^)	18.59 ± 2.10c	26.55 ± 0.16b	29.76 ± 0.41a	16.86 ± 0.19c
AvMn (mg·kg^−1^)	68.57 ± 0.74c	94.14 ± 1.40b	99.75 ± 0.65a	53.64 ± 0.58d
AvCu (mg·kg^−1^)	1.14 ± 0.01d	1.44 ± 0.01c	1.71 ± 0.02a	1.51 ± 0.03b
AvZn (mg·kg^−1^)	2.18 ± 0.06d	2.58 ± 0.03c	3.52 ± 0.04a	3.33 ± 0.03b
AvB (mg·kg^−1^)	0.57 ± 0.00c	0.62 ± 0.02c	0.83 ± 0.05b	0.93 ± 0.05a
AvMo (mg·kg^−1^)	0.17 ± 0.01c	0.23 ± 0.01a	0.24 ± 0.01a	0.20 ± 0.01b

Maize yield was assessed based on grain dry weight. SOM, soil organic matter; CEC, cation exchange capacity; AN, available nitrogen; AP, available phosphorus; AK, available potassium; ExCa, exchangeable calcium; ExMg, exchangeable magnesium; AvS, available sulfur; AvFe, available iron; AvMn, available manganese; AvCu, available copper; AvZn, available zinc; AvB, available boron; AvMo, available molybdenum. Values are presented as mean ± standard error (Mean ± SE, n = 3). Different lowercase letters within the same row indicate significant differences among treatments as determined by the Kruskal-Wallis test followed by Dunn’s multiple comparison test with Benjamini-Hochberg adjustment (*P* < 0.05). CK: no fertilization; NPK: chemical fertilizer; FNPK: NPK combined with farmyard manure; MNPK: NPK combined with bio-organic fertilizer.

**Figure 1:**
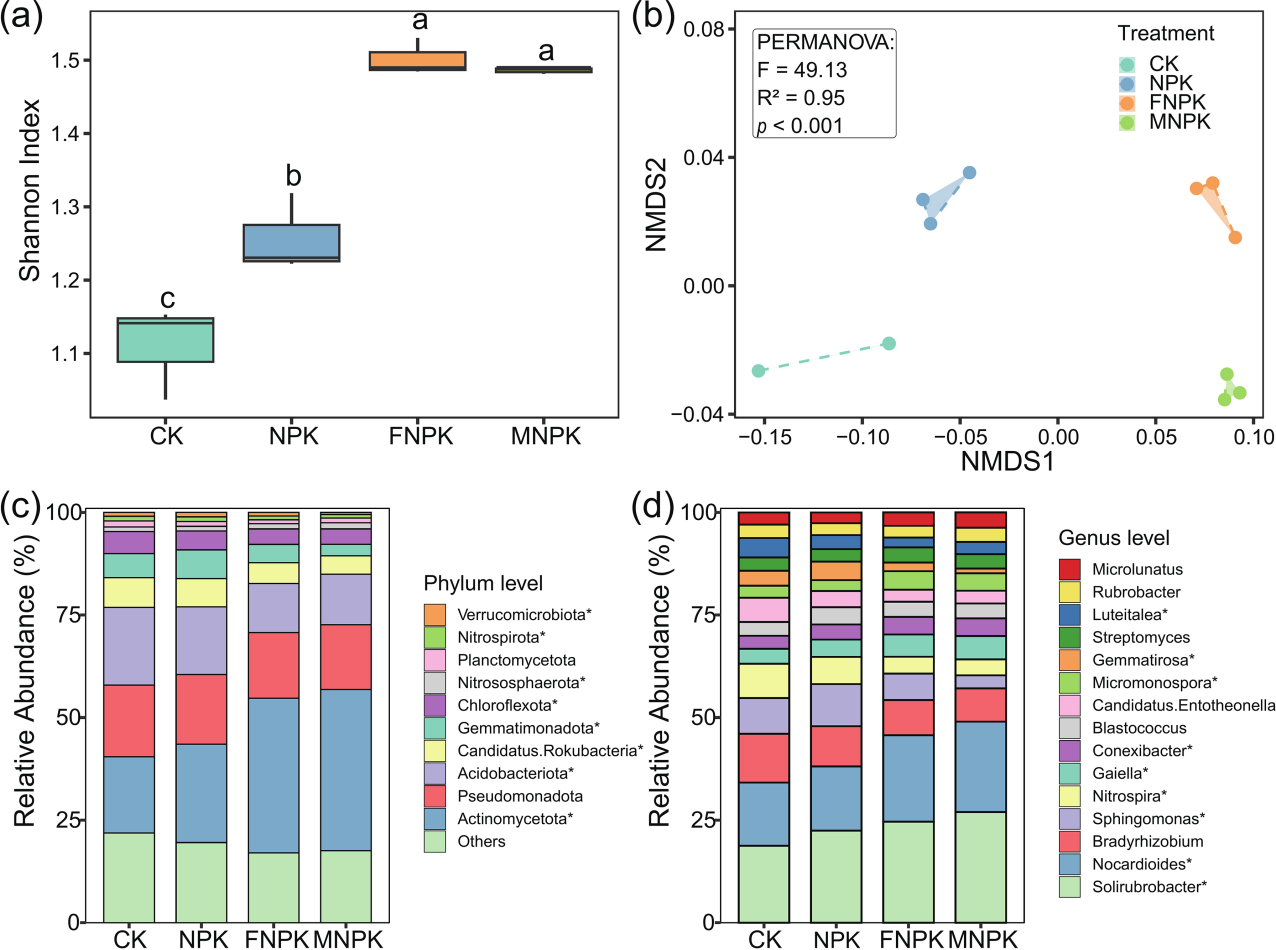
Effects of long-term fertilization on soil microbial diversity and community composition. **(a)** Shannon diversity index under different fertilization treatments; **(b)** NMDS, Non-metric multidimensional scaling ordination of microbial communities; **(c)** Relative abundance of microbial communities at the phylum level; **(d)** Relative abundance of microbial communities at the genus level. Asterisks indicate significant differences in relative abundance among the different treatments (*P* < 0.05).

Metagenomic analysis revealed that organic amendments fundamentally reshaped soil microbial diversity, community structure, and taxonomic composition (**Figure 1**). First, a significant increase in α-diversity (Shannon index) was observed in organically amended soils (FNPK and MNPK) compared to the mineral-fertilized (NPK) and control (CK) treatments (**Figure 1A**, *P* < 0.05). Second, β-diversity analysis via NMDS ordination, strongly supported by PERMANOVA (F = 49.13, R² = 0.95, *P* < 0.001), demonstrated a clear separation of microbial communities into distinct clusters corresponding to organic versus mineral fertilization regimes (**Figure 1B**). At the phylum level, organic amendments selectively enriched copiotrophic taxa such as Actinomycetota and Nitrososphaerota, while significantly (*P* < 0.05) depleting oligotrophic lineages including Acidobacteriota, Candidatus Rokubacteria, Gemmatimonadota, and Chloroflexota (**Figure 1C**). This selective restructuring was further evident at the genus level, where key functional groups like *Solirubrobacter*, *Nocardioides*, and *Gaiella* (Actinomycetota) were significantly (*P* < 0.05) enriched in FNPK and MNPK soils, whereas *Bradyrhizobium*, *Sphingomonas*, *Nitrospira*, and *Gemmatirosa* were markedly reduced (**Figure 1D**).

**Figure 2:**
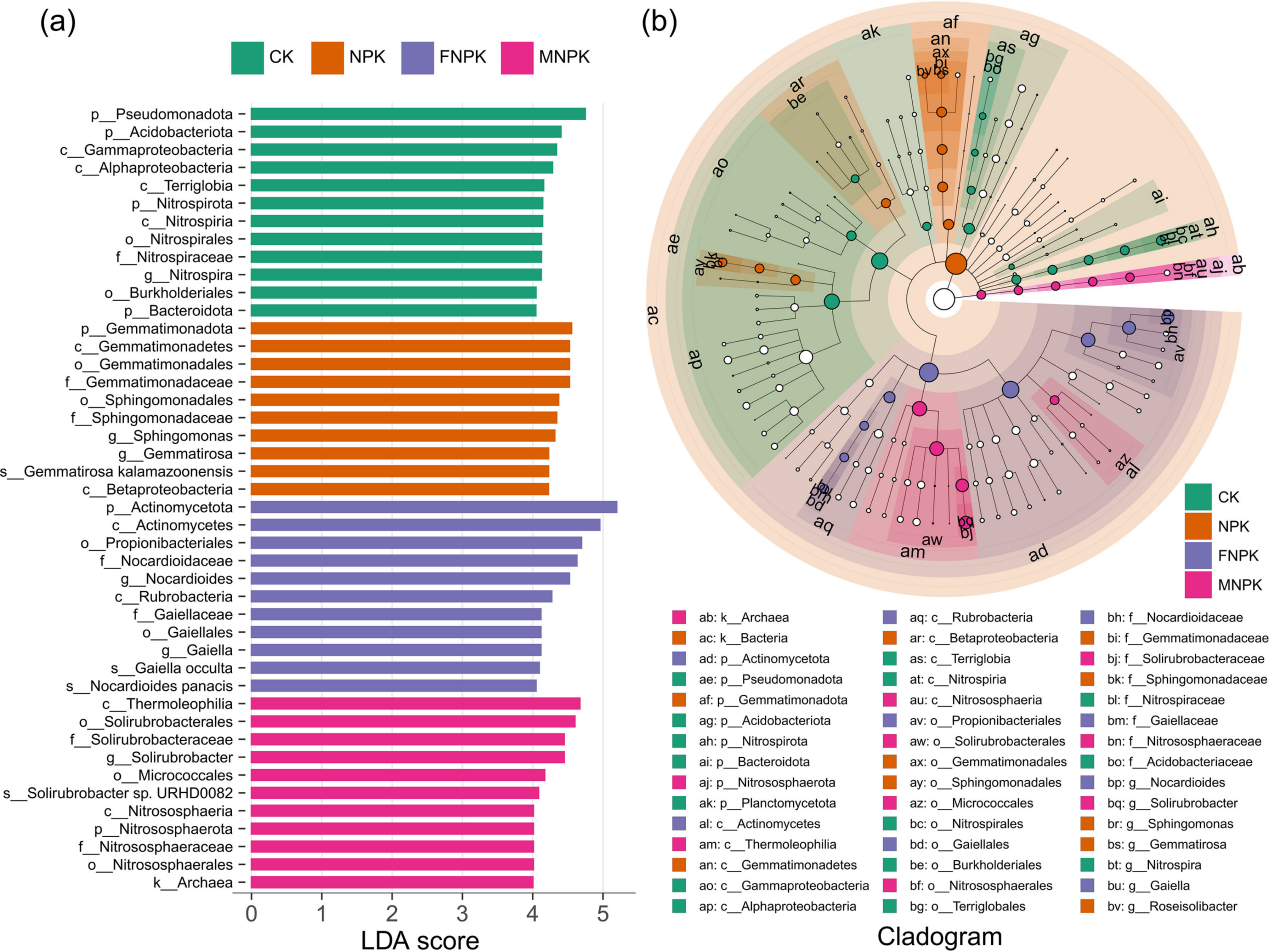
LEfSe-based differential taxonomic analysis of soil microbial communities. **(a)** Differential taxa identified by LEfSe analysis with an LDA score threshold of 4.0. **(b)** Cladogram illustrating the phylogenetic distribution of taxa showing significant differences among treatments.

LEfSe analysis identified treatment-specific microbial biomarkers (**Figure 2**). CK soils were enriched in taxa from the phyla Pseudomonadota, Acidobacteriota, and Nitrospirota, with the genus *Nitrospira* as a key feature. NPK soils were selectively enriched with Gemmatimonadota and the genus *Sphingomonas* (Pseudomonadota), a pattern indicative of adaptation to high mineral nutrient availability. FNPK strongly enriched Actinomycetota, particularly the genera *Nocardioides* and *Gaiella*, reflecting a consortium of taxa specialized in complex organic matter turnover. MNPK was characterized by the enrichment of Actinobacteria (notably the class Thermoleophilia and the genus *Solirubrobacter*) and archaea of the phylum Nitrososphaerota, suggesting a potential enhancement of ammonia oxidation processes.

### 3.2 Shifts in carbon, nitrogen, and phosphorus cycling pathways under different fertilization regimes

**Figure 3:**
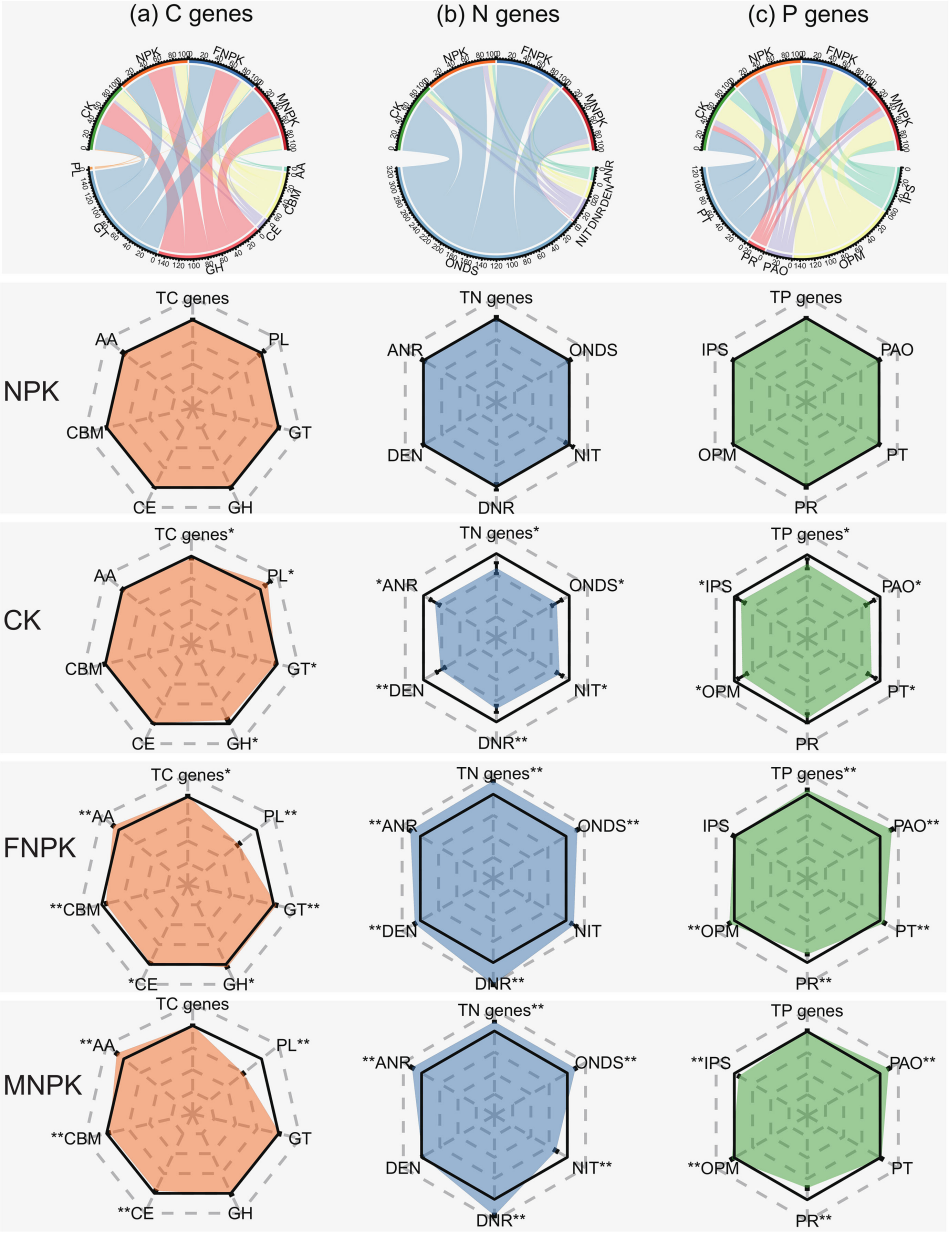
Relative abundances of functional genes involved in carbon, nitrogen, and phosphorus cycling under different fertilization treatments, and their differences relative to the NPK treatment. Asterisks (*) and double asterisks (**) on CK, FNPK, and MNPK bars indicate significant differences from NPK at *P* < 0.05 and *P* < 0.01, respectively. **(a)** Carbon cycling–related genes; **(b)** nitrogen cycling–related genes; **(c)** phosphorus cycling–related genes.

At the gene level, different fertilization regimes maintained the overall stability of biogeochemical cycling frameworks while significantly (*P* < 0.05) altering the composition of key functional pathways (**Figure 3**). The addition of organic amendments (FNPK and MNPK) induced a systematic reprogramming of these pathways across carbon, nitrogen, and phosphorus cycles. Regarding carbon cycling (**Figure 3A**), organic amendments significantly (*P* < 0.05) enriched genes for Auxiliary Activities (AA) and, in FNPK, Glycosyl Transferases (GT) and Glycoside Hydrolases (GH), which led to an increase in total carbon cycling genes. Concurrently, they reduced genes for Polysaccharide Lyases (PL), Carbohydrate-Binding Modules (CBM), and Carbohydrate Esterases (CE) compared to NPK, with PL decreasing by ~25%. For nitrogen cycling (**Figure 3B**), both organic amendments substantially increased the overall gene abundance and specifically enhanced assimilatory and reductive pathways—including Organic Nitrogen Degradation and Synthesis (ONDS), Assimilatory Nitrate Reduction (ANR), and Dissimilatory Nitrate Reduction (DNR)—while MNPK uniquely inhibited Nitrification (NIT). Concerning phosphorus cycling (**Figure 3C**), organic amendments consistently increased genes for Organic Phosphate Mineralization (OPM) and Phosphate-Accumulating Organisms (PAO) but suppressed those for Phosphate Regulation (PR). Notably, the amendments elicited type-specific effects: FNPK markedly enriched Phosphate Transport (PT) genes, whereas MNPK inhibited Inorganic Phosphate Solubilization (IPS). Consequently, FNPK significantly (*P* < 0.05) elevated the total phosphorus cycling gene pool compared to NPK, an effect not observed with MNPK.

### 3.3 Drivers of maize yield: integrative analysis of soil chemical, microbial, and functional gene properties

**Figure 4:**
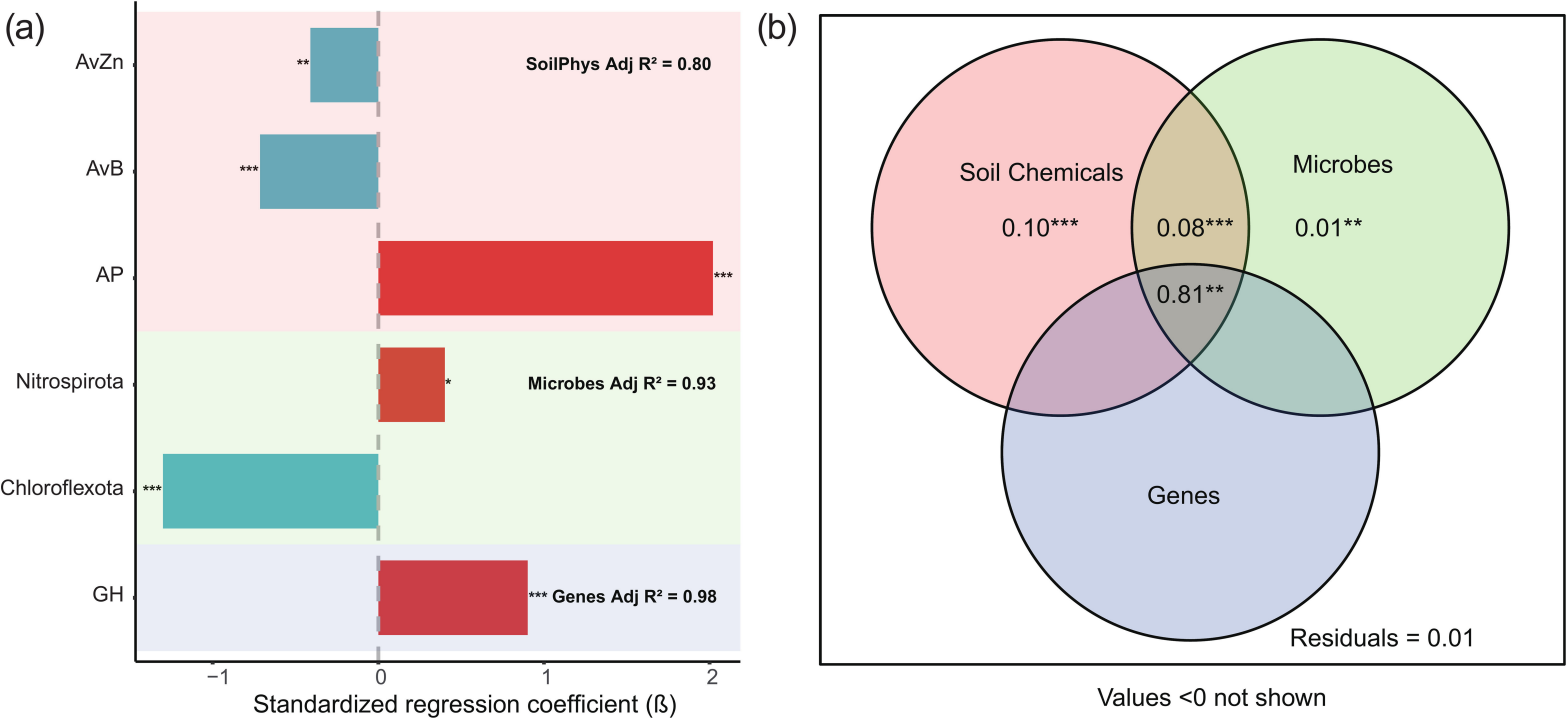
Stepwise regression and VPA, variance partitioning analysis of maize yield drivers. **(a)** Stepwise regression identified key factors within soil chemical properties, microbial phyla, and functional genes that significantly influence maize yield. **(b)** VPA of the selected key factors showing the independent and combined contributions of soil, microbial, and functional gene factors to yield variation.

Stepwise regression identified the key variables influencing maize yield within each factor group (**Figure 4A**): available phosphorus (AP), zinc (AvZn), and boron (AvB) for soil properties; the microbial phyla Nitrospirota and Chloroflexota; and the carbon-cycling gene family Glycoside Hydrolases (GH). These key factors were subsequently subjected to variance partitioning analysis (VPA) (**Figure 4B**). The VPA revealed that soil properties alone explained 10% of the yield variation, microbial community composition alone explained 1%, and functional genes alone had negligible explanatory power (0%). The two-way interaction between soil properties and microbial communities accounted for an additional 8% of the variation, whereas interactions involving functional genes contributed minimally. Most strikingly, the three-way interaction among all factor groups (soil × microbe × gene) explained the majority (81%) of the observed yield variation. This result underscores that neither functional genes nor microbial communities assessed in isolation are sufficient; rather, a holistic integration of soil chemical properties, microbial community structure, and functional gene potential is essential to accurately capture the primary determinants of maize yield.

**Figure 5:**
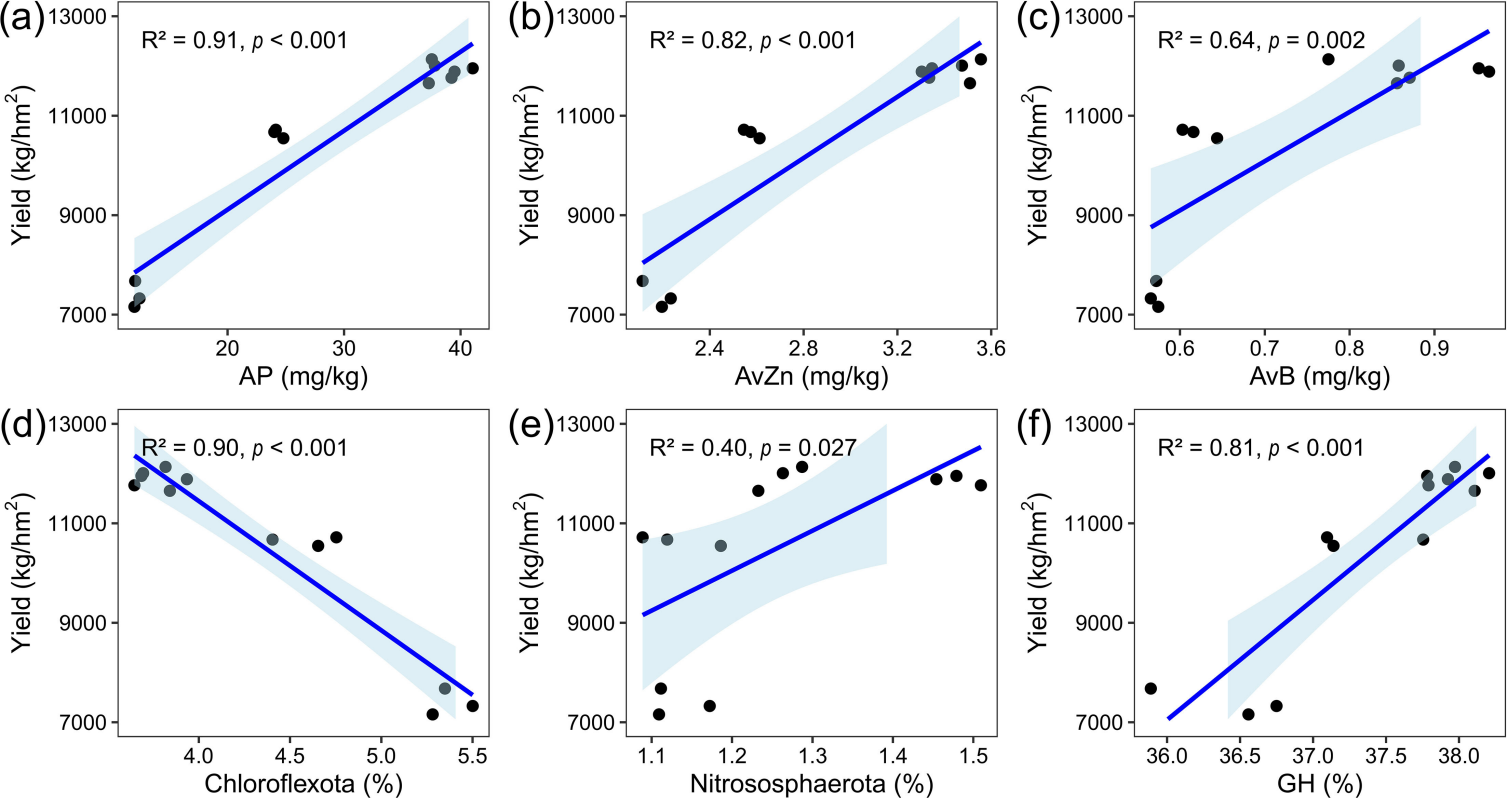
Linear relationships between maize yield and the key factors identified by stepwise regression. **(a)** AP vs. yield; **(b)** AvZn vs. yield; **(c)** AnB vs. yield; **(d)** Chloroflexota vs. yield; **(e)** Nitrososphaerota vs. yield; **(f)** GH vs. yield.

Linear regression analyses further corroborated the significant association of these key factors with maize yield (**Figure 5**). AP (r = 0.91), AvZn (r = 0.82), AvB (r = 0.64), Nitrospirota (r = 0.90), and GH (r = 0.81) exhibited strong positive correlations, whereas Chloroflexota showed a moderate negative correlation (r = -0.40).

**Figure 6:**
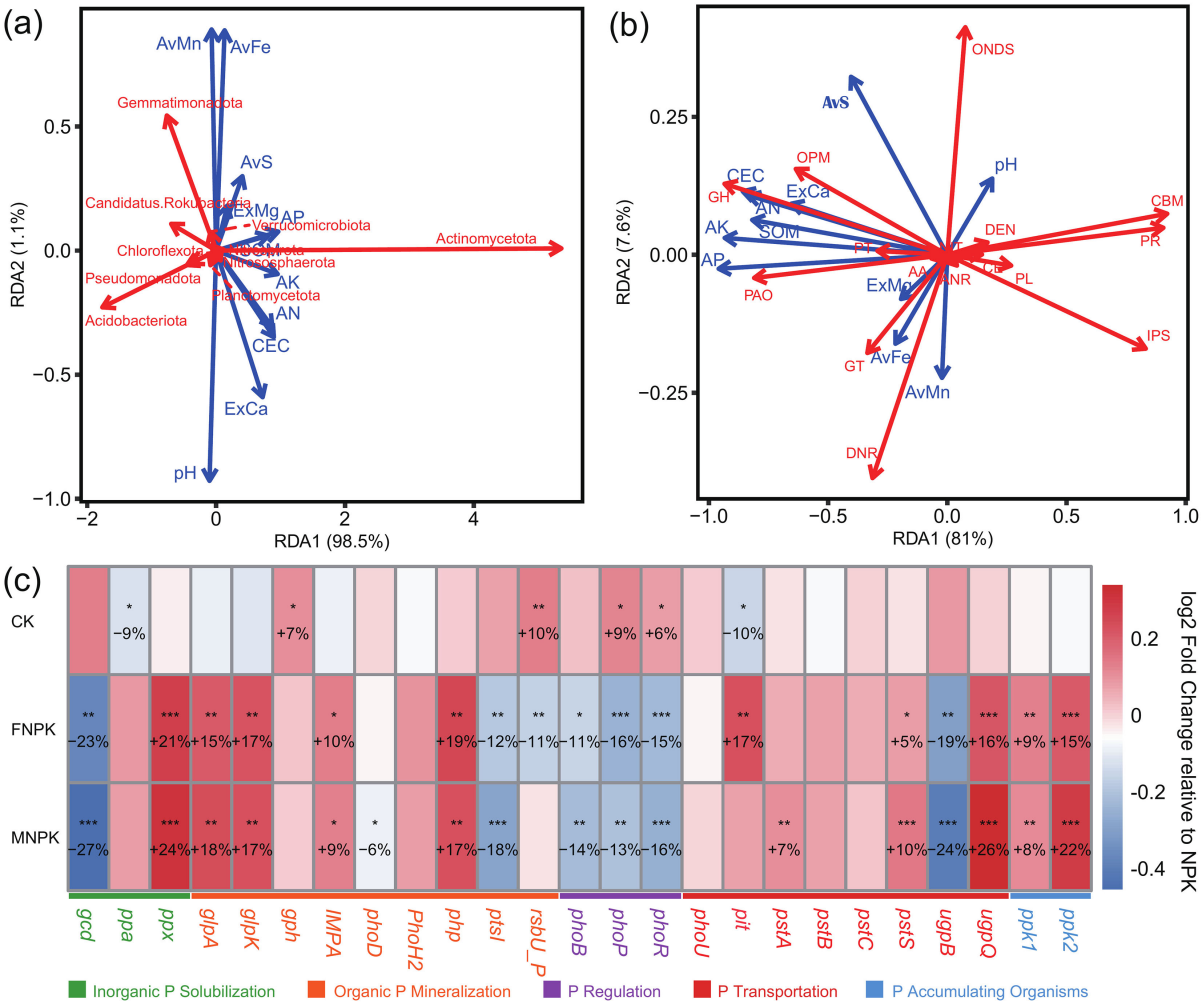
RDA, redundancy analysis showing the main drivers of microbial communities **(a)** and functional genes **(b)**, and comparison of phosphorus cycling genes among CK, FNPK, MNPK, and NPK treatments **(c)** log2 fold change relative to NPK). Asterisks indicate significant differences from NPK at *P* < 0.05 (*), *P* < 0.01 (**), and *P* < 0.001 (***).

Redundancy analysis (RDA) revealed that soil chemical properties were primary drivers of both microbial community structure and functional gene composition (**Figures 6A, B**). At the community level, RDA1 was predominantly influenced by available phosphorus (AP), available potassium (AK), cation exchange capacity (CEC), available nitrogen (AN), and soil organic matter (SOM), with pH primarily driving RDA2. Microbial phyla exhibited distinct responses: Actinomycetota was strongly associated with higher nutrient and SOM levels, whereas Acidobacteriota, Gemmatimonadota, and Pseudomonadota showed negative responses. At the functional gene level, the same suite of soil properties (AP, AK, CEC, AN, SOM) shaped the distribution of carbon, nitrogen, and phosphorus cycling genes along RDA1. Notably, phosphorus-related genes (e.g., CBM, PR, IPS) responded positively to these nutrient gradients, whereas Glycoside Hydrolases (GH, a carbon-cycling gene) responded negatively.

Given that AP was not only a key predictor of maize yield but also a central regulator in the RDA ordination, we conducted a targeted analysis of phosphorus cycling genes to elucidate the microbial mechanisms underpinning organic amendment effects (**Figure 6C**). Compared with the mineral-fertilized control (NPK), organic amendments (FNPK and MNPK) significantly (*P* < 0.05) upregulated a broad suite of genes involved in inorganic phosphate solubilization (*ppx*), organic phosphate mineralization (*glpA*, *glpK*, *IPMA*, *php*), phosphate transport (*pstS*), and polyphosphate metabolism (*ugpQ*, *ppk1*, *ppk2*), indicating a systemic enhancement of microbial phosphorus mobilization and storage capacity. The two organic amendments further induced distinct genetic strategies: FNPK upregulated the low-affinity transporter pit while downregulating *rsbU_P*, suggesting a shift toward direct phosphorus uptake; conversely, MNPK enriched the high-affinity transporter *pstA*, indicative of a high-efficiency phosphorus scavenging strategy. In stark contrast, the unfertilized control (CK) activated the phosphate stress response regulator *phoR*/*phoP*, reflecting a state of chronic phosphorus limitation.

### 3.4 Mechanisms of organic amendments effects on maize yield

**Figure 7:**
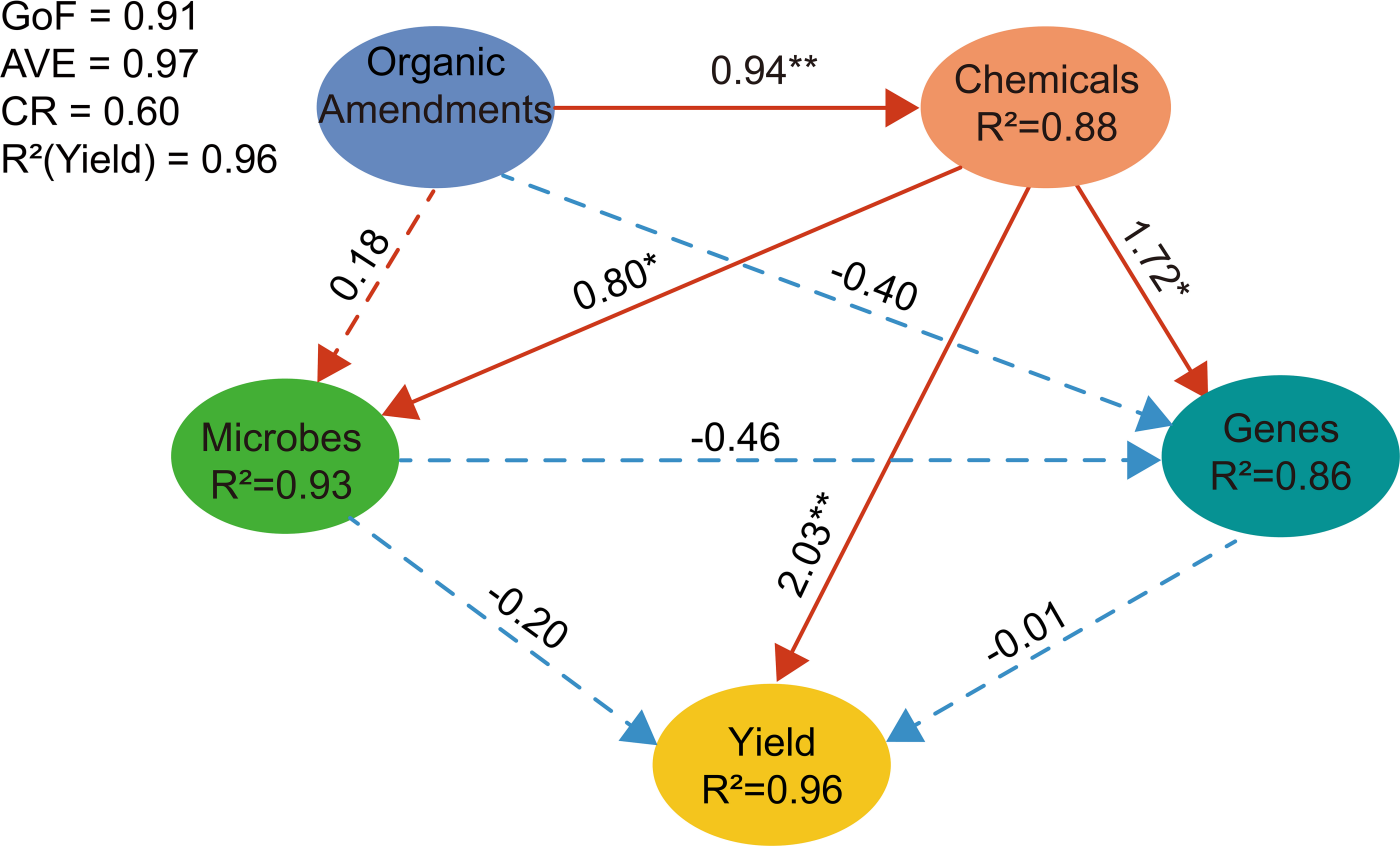
PLS-PM, partial least squares path modeling of the relationships among organic amendments, soil chemical properties, microbial communities, functional genes and maize yield. Red and blue arrows represent positive and negative effects, respectively, with numbers along the arrows indicating path coefficients. Significance levels are indicated as *P* < 0.05(*) and *P* < 0.01(**). Solid lines denote statistically significant pathways (*P* < 0.05), whereas dashed lines indicate non-significant pathways (*P* > 0.05). The model demonstrates excellent fit (GoF = 0.92) with strong convergent validity (AVE = 0.97) and explanatory power for maize yield (R² = 0.96). Composite reliability is 0.60.

Partial least squares path modeling (PLS-PM) was employed to elucidate the pathways through which organic amendments influence maize yield via the integrated soil–microbe–functional gene system (**Figure 7**). The model revealed that organic amendments exerted a strong, direct positive effect on soil chemical properties (path coefficient = 0.94, *P* < 0.01). In turn, soil properties exhibited a robust, direct positive effect on maize yield (path coefficient = 2.03, *P* < 0.01). In addition, soil properties were significantly associated with microbial community structure (path coefficient = 0.80, *P* < 0.05) and functional gene composition (path coefficient = 1.72, *P* < 0.05). However, neither microbial community structure nor functional gene composition exhibited a significant direct relationship with maize yield (*P* > 0.05).

## 4 Discussion

### 4.1 Restructuring the soil habitat through organic amendments

The long-term yield enhancement observed with organic amendments (FNPK and MNPK) reveals that their function in these phosphorus-deficient karst soils goes beyond mere nutrient supplementation. They primarily act as agents of soil habitat restructuring, first and most directly by modifying the chemical foundation of the soil (Salman et al., 2023). Compared to mineral fertilizer (NPK) alone, organic inputs significantly (*P* < 0.05) increased soil organic matter (SOM), cation exchange capacity (CEC), and the availability of the three major nutrients (AN, AP, AK) and micronutrients (Cu, Zn, B). Notably, the moderate increase in SOM under the mineral NPK treatment may be attributed to enhanced crop biomass production and greater root residue return to the soil following long-term fertilization (Ladha et al., 2011). In addition, improved plant growth under balanced NPK supply can stimulate rhizodeposition and microbial turnover, which indirectly contributes to SOM accumulation (Geisseler and Scow, 2014; Meng et al., 2024). The consistent improvement gradient (MNPK > FNPK > NPK) implies that bio-organic fertilizer, through its labile carbon and introduced *Bacillus* consortia, may more efficiently stimulate the formation of organo-mineral complexes, thus enhancing nutrient retention and bioavailability (Wortman et al., 2017; Wang et al., 2022).

Regulating soil pH represents a key aspect of this habitat change. The MNPK treatment specifically mitigated the acidification caused by mineral nitrogen, maintaining a near-neutral pH comparable to the unfertilized control (CK). This effect is attributed mainly to the input of basic cations and the organic anions produced during decomposition, which neutralize protons generated from nitrification (Wang et al., 2019), possibly assisted by associated microbial activity (Radhakrishnan et al., 2017; Qi et al., 2025). Conversely, farmyard manure (FNPK) did not significantly reduce acidification, indicating a material-specific response, likely due to a lesser capacity to generate buffering organic ligands (Meng et al., 2012). This pH divergence further governed micronutrient solubility, resulting in a distinct pattern. Iron and manganese availability was greater in the more acidic FNPK soil, while copper, zinc, and boron levels were elevated in both organic treatments through organo-metallic complexation (Cui et al., 2018; Hafez et al., 2025). Taken together, organic amendments first create a transformed chemical habitat—defined by higher nutrient status, greater buffering capacity, and an altered pH. This reconfigured habitat acts as the principal environmental filter that directs the assembly of the soil biological community.

### 4.2 Microbial community succession and functional gene expression under modified soil conditions

The chemical habitat reshaped by organic amendments precipitated a coherent shift in the soil microbiome, steering its transition from an oligotrophic to a copiotrophic state. Increased α-diversity and distinct β-diversity clustering under organic treatments confirmed that the addition of heterogeneous organic substrates alleviated carbon limitation—the primary constraint in these soils—and created varied ecological niches, thereby overriding spatial heterogeneity as the dominant force in microbial assembly (Chen et al., 2018; Lin et al., 2019). This directional change in community structure aligned with established ecological strategies: copiotrophic Actinomycetota (e.g., *Nocardioides*, *Gaiella*), which specialize in decomposing complex organic matter, were enriched, while oligotrophic Acidobacteriota, adapted to nutrient-poor and acidic conditions, declined (Hu et al., 2021; Liu et al., 2023; Zhou et al., 2024). The marked reduction in diazotrophic *Bradyrhizobium* and oligotrophic *Sphingomonas* further indicated that high nutrient availability relaxed the selection pressure for nitrogen fixation and survival under resource scarcity (Daims et al., 2015; Huang et al., 2021). Collectively, these patterns indicate that organic amendments induced a systematic restructuring of the soil microbiome toward a more diverse and functionally competent copiotrophic community, with enhanced potential for organic matter turnover and nutrient cycling.

These changes in microbial taxonomy were mirrored by corresponding adjustments in functional gene abundance, a relationship clearly illustrated by RDA showing strong correlations between key soil properties (AP, SOM, CEC) and both community structure and gene profiles (Jing et al., 2020; Philippot et al., 2024). At the genetic level, organic amendments consistently enhanced microbial capabilities for decomposing complex organic matter, as seen in the increased abundance of Auxiliary Activities (AA), Glycosyl Transferases (GT), and Glycoside Hydrolases (GH) alongside decreased Polysaccharide Lyases (PL) (Chen et al., 2025). For nitrogen cycling, both amendments enriched assimilatory (ANR) and reductive (DNR, ONDS) pathways while MNPK specifically suppressed nitrification genes (NIT), indicating a shift toward nitrogen conservation rather than loss-prone transformations (Geisseler and Scow, 2014; Li et al., 2015b). Most notably, phosphorus cycling genes revealed a fundamental functional divergence: although both treatments upregulated genes for organic P mineralization, transport, and storage (Xiao et al., 2021), FNPK preferentially enriched low-affinity, high-capacity phosphate transporters (*pit*) whereas MNPK induced high-affinity transporters (*pstA*). This pattern indicates a reinforced microbial strategy for decomposing and transforming complex organic matter.and further suggests that FNPK supports a “quantity-oriented” phosphorus acquisition strategy adapted to higher P availability, while MNPK fosters an “efficiency-oriented” scavenging approach under more competitive conditions—a distinction with important practical implications for managing different organic inputs.

### 4.3 A hierarchical pathway to yield enhancement and its practical implications

This study clarifies the pathway through which organic amendments enhance maize yield by integrating soil properties, microbial communities, and functional genes. The most revealing finding came from variance partitioning analysis (VPA), which demonstrated that while soil properties, microbial communities, or functional genes alone explained limited yield variation (10, 1% and 0%, respectively), their three-way interaction accounted for 81% of the observed variation. This result indicates that maize yield emerges from the synergistic interaction of these system components rather than from any single factor. In terms of productivity, yields under unfertilized control (CK, 7387 kg ha^-1^) were comparable to the national average for China in the corresponding year (National Bureau of Statistics of China, 2023), while conventional NPK fertilization (10645 kg ha^-1^) exceeded the national mean. Combined organic–mineral treatments (FNPK and MNPK) further increased yields to 11931 kg ha^-1^ and 11868 kg ha^-1^, respectively. The generally high yields across all treatments are likely associated with the relatively high soil organic matter at the experimental site, but the additional gains under FNPK and MNPK clearly demonstrate the effectiveness of organic amendments in enhancing maize productivity beyond mineral fertilization alone.Partial least squares path modeling (PLS-PM) further delineated the structure of this synergy. The model confirmed that organic amendments primarily influence yield by first modifying soil chemical properties (path coefficient = 0.94), which then directly and strongly affect yield (path coefficient = 2.03). These altered soil conditions serve as the main driver of both microbial community structure and functional gene composition. Crucially, no significant direct path existed from microbes or genes to yield. This does not diminish the biological role but rather clarifies it: microbial communities and their functional genes act as essential intermediaries that translate improved soil conditions into more efficient nutrient cycling (Ren et al., 2023; Pandey and Saharan, 2025). Their contribution is ultimately captured within the “soil property” variable that directly influences plant growth. This aligns with the central role of available phosphorus (AP)—identified here as a key yield determinant and the primary driver in RDA—in regulating microbial function in phosphorus-limited karst ecosystems (Vitousek et al., 2010; Wang et al., 2024).

Therefore, in karst yellow soils, organic amendments improve yield through a nutrient-driven hierarchical process. Amendments first enhance the soil’s chemical habitat, which in turn selects for a more active, functionally specialized microbiome. This adapted microbial community then executes more efficient nutrient cycling—particularly phosphorus mobilization, potentially augmented by specific microbes like *Bacillus* in MNPK (Li et al., 2023; Iqbal et al., 2024). These interconnected changes collectively establish the high nutrient availability that directly supports maize production. This understanding moves beyond simple questions of whether microbes or nutrients drive yield. Instead, it supports a management approach that prioritizes creating favorable soil conditions as the essential first step, recognizing that a functionally effective microbial community will subsequently develop to optimize nutrient dynamics within that improved environment.

## 5 Conclusions

Our study demonstrated that organic amendments enhance maize yield in karst soils through a soil-centered hierarchical mechanism. Rather than directly regulating microbes or functional genes, these inputs first improve the chemical properties of soil, particularly by increasing phosphorus availability. This enhanced soil environment subsequently promotes crop yield directly while also shifting the microbial community toward a copiotrophic state and upregulating functional genes related to nutrient cycling, such as those for organic phosphorus mineralization. Statistical path modeling confirms that these microbial and genetic responses are outcomes of soil improvement, not direct drivers of yield, establishing a clear causal sequence from amendments to soil properties to productivity. Therefore, in phosphorus-fixing karst agroecosystems, organic amendments should be primarily regarded as soil conditioners. Sustainable management must focus first on improving soil health, which then naturally fosters a functionally adapted microbial community that supports efficient nutrient cycling and stable crop production.

## Data Availability

The datasets presented in this study can be found in online repositories. The names of the repository/repositories and accession number(s) can be found below: https://ngdc.cncb.ac.cn/search/specific?db=bioproject&q=PRJCA050203, PRJCA050203.
